# Editorial: Emerging technologies in occupational health and safety

**DOI:** 10.3389/fpubh.2023.1117396

**Published:** 2023-02-07

**Authors:** Hamzeh Mohammadi, Hadiseh Rabiei, Somayeh Farhang Dehghan

**Affiliations:** ^1^Faculty of Health and Medical Engineering, Tehran Medical Sciences, Islamic Azad University, Tehran, Iran; ^2^Student Research Committee, Department of Occupational Health and Safety at Work, School of Public Health and Safety, Shahid Beheshti University of Medical Sciences, Tehran, Iran; ^3^Environmental and Occupational Hazards Control Research Center, School of Public Health and Safety, Shahid Beheshti University of Medical Sciences, Tehran, Iran

**Keywords:** occupational health and safety, emerging technologies and practices, risk management, work stress, anti-UV, driving fatigue

Occupational health and safety (OHS) is a field devoted to the anticipation, recognition, evaluation, and control of these environmental factors or stresses arising in or from the workplace. The application of technology is considered as an effective approach to improve workers' health and safety conditions and ensure health and safety management in general. Research and development is directed toward the advancement of technology, and therefore includes the development of emerging technologies. Emerging technologies (e.g., artificial intelligence, nanotechnology, virtual reality and robotics) are characterized by radical novelty, relatively fast growth, coherence, prominent impact, and uncertainty and ambiguity. Emerging technologies in occupational health and safety are those technical innovations which represent progressive developments within the different fields of OHS (from the point of view of hazardous agents like chemical, physical, biological, ergonomics and safety) in different phases of risk management including identifying, assessing and controlling the risks. Emerging technologies offer challenges and opportunities with regard to worker safety and health. Although the emergence of novel technologies could provide a lasting solution to the problem of workplace health and safety, it is necessary to understand and prevent any hazards arising from them. The scope of this special Research Topic is to introduce the emerging technologies and their practical applications in anticipation, recognition, evaluation, and controlling the hazardous agents including chemical, physical, biological, ergonomics and safety at the workplace. Moreover, it aims at reviewing and assessing the hazards of emerging technologies which may limit their success and offer designs that eliminate the hazards of emerging technologies.

The Editorial aimed to present the contributing articles of the Research Topic related to Section Occupational Health and Safety of Frontiers in Public Health in 2022. The Research Topic has published articles from February to August regarding the use of emerging technologies in assessing workplace stress, preventing chemotherapy agent contamination, detecting driving fatigue, use of e-consultations during the COVID-19 pandemic, implementing a chemical risk assessment, developing an UV protective fabric and building a modified goggle. Also, the studies were conducted in countries such as China, Saudi Arabia, and Iran.

Li et al. assessed workplace stress among nurses using heart rate variability analysis and wearable ECG. They asked 17 nurses from a major public hospital in China to wear an ECG monitor to measure stress level during work, and to complete the Chinese Nurses Stress Response Scale after work as subjective response criteria. They demonstrated that it is feasible to use wearable ECG devices and heart rate variability (HRV) analysis to investigate workplace stress under work conditions in nurses. They also indicated that this approach may serve as a preventive measure for identifying stress-related illnesses in nurses.

In the risk management of OHS, the use of wearable ECG can be useful to investigate work stress and mental workload and also, early identification of at-risk individuals of cardiovascular disorders. HRV analysis can also be used to estimation physical activity levels and energy metabolism, which are two important parameters in assessing heat stress and ergonomic issues.

Tang et al. did a comparative study to evaluate closed system transfer devices (CSTDs) in preventing chemotherapy agents contamination during compounding process and investigate it's their efficacy on the safe management of injectable hazardous drugs (HDs). The exposure assessments of cyclophosphamide and cytarabine were performed under traditional or CSTDs. A total of 96 wiping samples from protective equipment (such as gloves and masks) were collected and the contamination analysis was performed by liquid chromatography with tandem mass spectrometry. They indicated that the proper use of a CSTD may significantly decrease contamination by these drugs and consequently decrease exposure risk on workplace surfaces and personal protective equipment as compared to traditional compounding devices. They concluded that CSTDs are offering progressively more effective alternatives to traditional ones and consequently decrease chemotherapy exposure risk on isolator surfaces.

The topic of measures taken to protect handlers from occupational exposure to chemotherapy has always been controversial. Although work experience can be very effective in the level of exposure to it, in the case of some chemicals, exposure to a low concentration can lead to unpleasant consequences. The use of the isolated unit, which can greatly reduce the possibility of respiratory or skin exposure, is necessary in these high-risk worksites.

Rabiei et al. evaluated the ultraviolet (UV) protective factor (UPF) of workwear fabrics coated with the TiO_2_ nanoparticles (NPs) prepared using an *in-situ* synthesis method. They concluded that the UV protective properties of fabrics can be improved by coating the TiO_2_ NPs on them without any significant effect on the intrinsic properties of fabrics. However, they indicated that future studies can evaluate the cytotoxicity properties of the TiO_2_ NPs to ensure that they have no adverse effects on human skin.

Occupational exposure to UV radiation can occur in many outdoor and indoor workplaces, like welding, healthcaring, laboratories, farming and mining, which can cause skin and eye damage, so that the use of anti-UV clothing can be considered as a preventive measure.

Fatemi et al. developed and implemented a risk assessment method to determine and prioritize hazardous chemicals in academic laboratories. The study was conducted at five academic laboratories and research facilities of a Medical Sciences University in Iran. They reported the adequate safety provisions and procedures in the laboratory operations and also, they found that the lack of awareness concerning health, safety, environmental chemical hazards, and inappropriate sewage disposal systems contributed to the increasing levels of laboratory risk. They suggested the need for improving the risk perception of individuals involved in handling chemicals to prevent exposure to workplace duties and environmental pollution hazards.

Doing the chemical risk assessment in work environments can have different applications, such as:

- Identification of the risks related to all chemicals that are handled, stored or transported,
- Exposure evaluation of individuals to hazardous chemicals,
- Identification of tasks that have a high health and safety risk,
- Assessing the adequacy of available control measures, and
- Adopting the appropriate control measures to eliminate or reduce risk.

Althumairi et al. identified the factors that influence current patient use and the intention of using e-consultation in Saudi Arabia. A cross-sectional survey was distributed online *via* social media platforms targeting the population living in Saudi Arabia from August to December 2020. A total of 150 participants completed the questionnaire. They concluded that participants' trust in and perception of the usefulness of e-consultations were significant factors in their intention to use e-consultation services. They indicated that policymakers' attention to those factors could play a role in increasing public acceptance and the use of e-consultations to improve medical care distance.

One of the most important factors affecting the spread of pandemics is the presence of carriers in crowded places. One of the solutions that can prevent such gatherings in places like hospitals or medical centers is providing remote and electronic services in non-emergency cases, which is considered as a management solution and administrative control measure in occupational health and safety.

Shi et al. assessed a combination of automated pupillometry and heart rate variability to detect driving fatigue. A 90-min monotonous simulated driving task was utilized to induce driving fatigue. During the task, measurements of pupillary light reflex were performed and subjective rating scales and heart rate variability were monitored simultaneously. They concluded that pupillary light reflex variation may be a potential indicator in the detection of driving fatigue, achieving a comparative performance compared with the combination with heart rate variability. They suggested that further work may be involved in developing a commercialized driving fatigue detection system based on pupillary parameters.

The fatigue caused by driving for long periods can affect driver concentration and performance and so jeopardize transportation safety. Predicted driver fatigue and providing warning alarms can make the driver more alert, which is a very important and preventing factor in road traffic crashes.

Shao et al. built a modified goggle (MG) with better physical performance and they used the temperature-humidity index (THI), an indicator to investigate the impact of goggle-related heat strain on the ocular surface. The basic functions of antifog, anti-ultraviolet (UV), and anti-blue-light radiation capabilities were evaluated. They also assessed the clinical impact on non-invasive keratography tear film break-up time (NIKBUT), intraocular pressure, central corneal thickness, Schirmer test I, and the Dry Eye-related Quality of life Score (DEQS) in 40 healthcare workers by comparing MG with standard goggles (SG). They found that wearing goggles for a long time may cause heat strain to the eyes, thereby leading to eye discomfort and changes in the microenvironment of the ocular surface. Their MG exhibited better antifog, antiultraviolet, and optimal anti-blue-light performance and lower heat strain than SG, thus making it ideally suited for healthcare workers.

During the outbreak of the COVID-19 pandemic, the use of goggles became common among healthcare workers, because one of the ways of transmission of the virus is through the mucous membrane of the eyes. Fogging of goggles can adversely affect the quality of medical work and also increase medical errors (such as needle sticks). The fogging of goggles is often because of the leakage of expired air into it and can result in ergonomic issues due to awkward posutres of healthcare workers like bending, in order to increase precision and visibility. Therefore, the use of antifog goggles, can increase job satisfaction and reduce medical errors and prevent musculoskeletal disorders.

Finally, by reviewing all the articles published in the Research Topic, it can be concluded that the use of emerging technologies (e.g., digitization; novel technology; nanomaterials) in occupational health and safety in many different fields (e.g., Anti-UV fabrics; antifog, anti- UV and anti-blue-light radiation goggles; developed tools for assessing work fatigue and stress) can be possible; from point of view of the development of tools to the designing and manufacturing products related to OHS issues.

## Author contributions

All authors listed have made a substantial, direct, and intellectual contribution to the work and approved it for publication.

